# Fetal Anomalies Associated with Novel Pathogenic Variants in *TMEM94*

**DOI:** 10.3390/genes11090967

**Published:** 2020-08-20

**Authors:** Mohamed H. Al-Hamed, Nada Alsahan, Maha Tulbah, Wesam Kurdi, Wafa’a I. Ali, John A. Sayer, Faiqa Imtiaz

**Affiliations:** 1 Genetics Department, King Faisal Specialist Hospital and Research Centre, P.O. Box 3354, Riyadh 11211, Saudi Arabia; Hamed@kfshrc.edu.sa; 2 Saudi Diagnostics Laboratory, KFSHI, P.O. BOX 6802, Riyadh 12311, Saudi Arabia; wafaa.ia@hotmail.com; 3 Department of Obstetrics and Gynecology, King Faisal Specialist Hospital and Research Centre, P.O. Box 3354, Riyadh 11211, Saudi Arabia; nalsahan@kfshrc.edu.sa (N.A.); mtulbah@kfshrc.edu.sa (M.T.); kurdi@kfshrc.edu.sa (W.K.); 4 Translational and Clinical Research Institute, Faculty of Medical Sciences, Newcastle University, Central Parkway, Newcastle upon Tyne NE1 3BZ, UK; john.sayer@newcastle.ac.uk; 5 Renal Services, The Newcastle Hospitals NHS Foundation Trust, Newcastle upon Tyne NE7 7DN, UK

**Keywords:** IDDCDF, *TMEM94*, pathogenic variant, prenatal exome, consanguinity

## Abstract

Background: Intellectual developmental disorder with cardiac defects and dysmorphic facies (IDDCDF, MIM 618316) is a newly described disorder. It is characterized by global developmental delay, intellectual disability and speech delay, congenital cardiac malformations, and dysmorphic facial features. Biallelic pathogenic variants of *TMEM94* are associated with IDDCDF. Methods and Results: In a prenatal setting, where fetal abnormalities were detected using antenatal sonography, we used trio-exome sequencing (trio-ES) in conjunction with chromosomal microarray analysis (CMA) to identify two novel homozygous loss of function variants in the *TMEM94* gene (c.606dupG and c.2729-2A>G) in two unrelated Saudi Arabian families. Conclusions: This study provides confirmation that *TMEM94* variants may cause IDDCDF. For the first time we describe the pathogenicity of *TMEM94* defects detected during the prenatal period.

## 1. Introduction

Neurodevelopmental disorders are genetically and phenotypically heterogeneous conditions and can be accompanied by congenital heart defects and dysmorphic features. Intellectual developmental disorder with cardiac defects and dysmorphic facies (IDDCDF, MIM 618316) is a recently described neurodevelopmental disorder, which is characterized by global developmental delay, intellectual disability and speech delay, congenital cardiac malformations, and dysmorphic facial features [1]. It was initially reported in ten patients from six unrelated families with an autosomal recessive pattern of inheritance. The patients ranged in age from 3 to 24 years, and most of them were of Middle Eastern descent. All affected patients had biallelic loss of function variants in the *TMEM94* gene (MIM 618163). *TMEM94* was mapped to chromosome 17q25.1 [2] and consisted of 31 coding exons (RefSeq NM_014738), which encoded for an uncharacterized transmembrane nuclear protein. In a murine model, homozygous loss of *TMEM94* was embryonically lethal and led to craniofacial defects, cardiac abnormalities, and abnormal neuronal migration in the central nervous system that mimicked human phenotype [1]. To date, all of the seven mutations in *TMEM94* were predicted to result in truncated proteins lacking the highly conserved C-terminal domain and showed significantly decreased *TMEM94* expression [1].

The combination of antenatal ultrasound scanning (USS) and molecular genetic testing is a powerful approach to identify the genes responsible for fetal abnormalities [3]. In some regions, the rate of consanguineous marriage in the Saudi Arabian population exceeds 55%, and the majority of genetic diseases are inherited in an autosomal recessive manner [4].

Here, we report two separate Saudi Arabian consanguineous families with novel loss of function variants in the *TMEM94* gene, presenting and detected in the prenatal period. The identification of the *TMEM94* mutations by this study confirms its role in IDDCDF and its identification antenatally. 

## 2. Materials and Methods 

Fetal blood and amniotic fluid samples were collected from 2 fetuses in addition to parental peripheral blood samples from two separate Saudi Arabian consanguineous families, diagnosed by antenatal sonography with fetal abnormalities. Families were diagnosed at the Maternal Fetal Medicine (MFM) section at the King Faisal Specialist Hospital and Research Centre (KFSH&RC). Written informed consent was obtained from families and the project was approved by the Research Advisory Council at the KFSH&RC (RAC# 2160 022). DNA was extracted using the Gentra Systems PUREGENE DNA Isolation kit (Qiagen, Germantown, MD, USA). Maternal blood contamination was excluded in all fetal samples by using the AmpFLSTR^®^ Identifiler^®^ PCR Amplification Kit (Applied Biosystems, Thermo Fisher Scientific, Foster City, CA, USA), as described by the manufacturer. Exome sequencing (ES) was performed using genomic DNA. Trio-ES was performed using an Agilent Sureselect All Exons V5 (50 Mb) capture kit (Agilent Technologies, Santa Clara, CA, USA), as described previously [5]. For variant interpretation, an in-house variant interpretation pipeline was used, as previously described [6]. Segregation studies and validation of all detected ES variants was confirmed by Sanger sequencing. Chromosomal microarray analysis (CMA) was performed to rule out chromosomal abnormalities in fetuses. For CMA, genomic DNA were fragmented, amplified, and hybridized to the array, according to manufacturer’s guidelines (Affymetrix CytoScan^®^ HD Array Kit, Thermo Fisher Scientifi, Foster City, CA, USA). It contains 2.7 million markers across the whole genome covering 96% of the genes. The results were analyzed with the Chromosome Analysis suite (ChAS, Affymetrix, Thermo Fisher Scientific, Foster City, CA, USA). Copy number variations (CNVs) with more than 50 kb (deletions) and 200 kb (duplications) were reported. Segregation studies and validation of all detected ES variants were confirmed by Sanger sequencing as described before [7].

## 3. Results and Discussion

**Figure 1:**
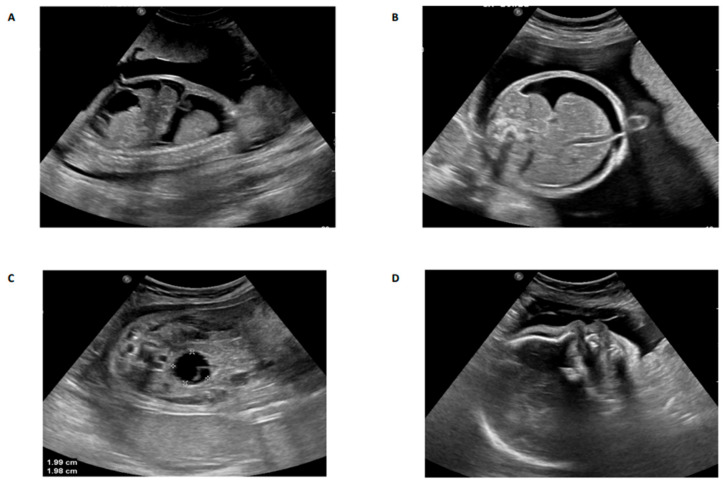
Antenatal ultrasound images of affected cases in family 1 (**A**,**B**) and family 2 (**C**,**D**). (**A**) Sagittal view of the fetal thorax and abdomen at 26 weeks of gestation, showing severe pleural effusion and ascites; (**B**) A transverse view of the fetal abdomen at 26 weeks of gestation showing ascites; (**C**) Coronal view of the fetal abdomen and pelvis at 29 weeks of gestation showing a well circumscribed hypoechoic area in the fetal abdomen which may represent an abdominal cyst with unknown origin; (**D**) Sagittal view of the fetal face at 29 weeks of gestation showing an abnormal profile with micrognathia.

**Figure 2:**
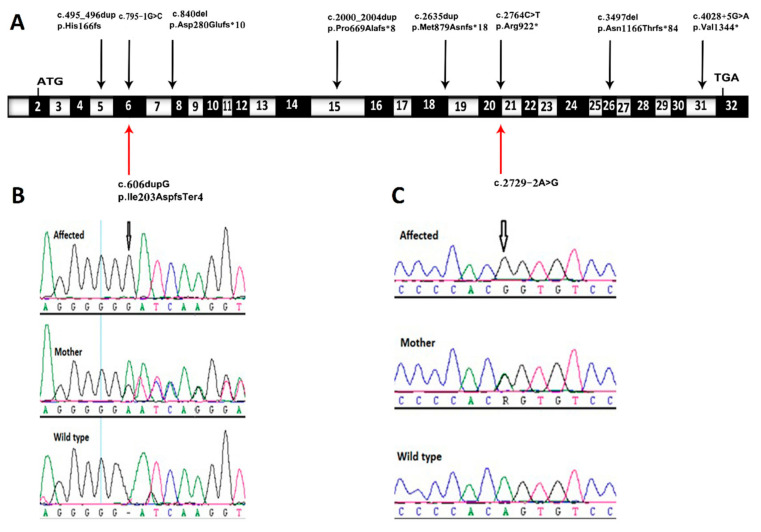
Sequence chromatograms demonstrating novel variants detected and their locations in *TMEM94* gene. (**A**) Schematic representation of exon structure of the *TMEM94* gene, which is showing identified mutations. The location of c.606dupG and c.2729-2A>G mutations are shown by red arrows; (**B**,**C**) Sequencing chromatograms of the affected fetus, parent, and wild-type normal control of family 1 (**B**) and family 2 (**C**) in the *TMEM94* gene (RefSeq NM_014738).

Two consanguineous families were referred to the MFM section at the KFSH&RC due to fetal anomalies in recent pregnancies. Using trio whole exome sequencing (trio-ES), in addition to chromosomal microarray analysis (CMA), a likely molecular genetic diagnosis was established in both families. Two homozygous loss of function sequence variants were detected in *TMEM94,* which were associated with early fetal anomalies phenotypes. Both sequence variants detected in *TMEM94* were novel. All sequence variants detected in these families were germline inherited variants and the parental segregation was confirmed by Sanger sequencing. A detailed history and analysis of the results for each family is given below as follows:

Family 1 this family was a first-degree consanguineous marriage from the Northern province of Saudi Arabia. The mother was 30 years old and had a prior history of four pregnancies. The first and second pregnancies were both full term by cesarean section, and both children are alive and well. The third pregnancy resulted in a miscarriage during the first trimester. The fourth and most recent pregnancy was referred to the KFSH&RC at 26 weeks of gestation due to the suspicion of fetal abnormalities. Fetal ultrasound scanning indicated generalized skin edema, ascites, unilateral hydrothorax, and polyhydramnios (Figure 1A,B). Fetal echocardiography was unremarkable. Cordocentesis was performed and samples were tested for Trio-ES and CMA. A thoracoamniotic shunt was placed to drain the pleural effusion. The child was delivered at 28 weeks by cesarean section due to severe preeclampsia in the mother. The child died immediately after delivery. The CMA result was negative for chromosomal abnormalities whilst Trio-ES detected a novel homozygous splice site mutation in the fetus (*TMEM94*: NM_014738.6: exon21: c.2729-2A>G) which was present in its heterozygous state in both parents (Figure 2). 

Family 2 this family was a first-degree consanguineous marriage from the Northern Province of Saudi Arabia. At diagnosis, the mother was 33 years old with two previous pregnancies. The first pregnancy was full-term with a successful outcome. The second pregnancy ended with spontaneous miscarriage during the first trimester. During her third pregnancy, she was referred to the MFM at the KFSH&RC following the identification of a thick nuchal fold in the fetus at 29 + 3 weeks. Antenatal ultrasound scanning confirmed a thick nuchal fold, as well as micrognathia, bilaterally clenched hands, and an abdominal cyst measuring 22 × 22 × 22 mm (Figure 1C,D). Fetal echocardiography showed a large aneurysmal atrial septal defect and small apical muscular ventricular septal defects. Amniocentesis was performed and samples were tested for Trio-ES. The CMA result was negative for chromosomal abnormalities, whereas Trio-ES detected a novel homozygous frameshift mutation in exon 6 of the *TMEM94* gene (*TMEM94*: NM_014738.6: exon6: c.606dupG: p.Ile203AspfsTer4) of the fetus, and both parents were heterozygous carriers (Figure 2). The baby was born at term at the local hospital with dysmorphic facial features, and birth weight around 4 kg (greater than normal for gestational age). The baby was transferred to the KFSH&RC but died at 6 months of age following cardiac surgery due to sepsis. A further pregnancy prompted referral to the KFSH&RC for prenatal diagnosis. Antenatal ultrasound scanning showed nuchal translucency. Chorionic villus sampling was performed at 13 weeks and six days, and the sample was tested for targeted mutational analysis. The pregnancy was terminated as the fetus was found to be homozygous for the familial pathogenic *TMEM94* mutation.

In humans, the transmembrane protein 94 is a multipass nuclear membrane protein encoded by the *TMEM94* gene. The uncharacterized protein contains 1356 amino acids and is expressed in all human tissues, with the highest levels in skeletal muscle and testis [2]. Although the phenotype associated with the defects in *TMEM94* was recently reported, the defect in another transmembrane protein, TMEM260 was reported to be phenotypically related with neurodevelopmental and cardiac anomalies [8]. It is well known that gene defects in many members of the transmembrane protein family, such as TMEM138 [9], TMEM165 [10], TMEM231 [11], TMEM237 [12], TMEM67 [13], and TMEM5 [14] are associated with fetal anomalies. *TMEM94* defects have been reported with a prenatal history of omphalocele, atrioventricular septal defect (AVSD), and intestinal malrotation, which was surgically corrected after birth in a Turkish patient aged 24 years of age [1]. The previous report also included three families with Arabian ancestry (from Oman, Qatar, and Egypt). In this report, we add two more families from Saudi Arabia to the cohort of affected families with *TMEM94* mutations. Cardiac defects (including pleural effusions), abdominal abnormalities (ascites and cysts), skeletal abnormalities (clenched hands), and dysmorphic features (micrognathia) are the main USS phenotypic findings in the reported families. The homozygous loss of function mutations c.606dupG and c.2729-2A>G within *TMEM94,* identified here, are novel (absent from the 1000 Genomes Project, Exome Sequencing Projects, Exome Aggregation Consortium, and Genome Aggregation Database), and were not found in our local in-house Saudi Arabian exome database. The pathogenic variant c.606dupG: p.Ile203AspfsTer4 in exon 6 of *TMEM94* is the most severe truncating mutation reported so far, leading to a premature stop codon at amino acid 206. The early premature truncation may correlate to severe clinical phenotype in the family. In both families, cardiac abnormality was the obvious structural irregularity identified by antenatal ultrasound. The loss of function variants detected in these two fetuses are correlated to the cardiac abnormality phenotype observed in the mouse model [1]. The identification of these two novel mutations increases the spectrum of pathogenic variants in *TMEM94* that associated with IDDCDF. In conclusion, we provide confirmation that *TMEM94* mutations lead to IDDCDF, and describe phenotypes associated with *TMEM94* defects presenting and diagnosed within the prenatal period.
